# Linking suspects to an illegally felled incense tree (*Aquilaria sinensis*) using DNA forensics

**DOI:** 10.1016/j.fsisyn.2026.100721

**Published:** 2026-08-11

**Authors:** Kerry Reid, Uva Y.Y. Fung, Arthur F. Sands, Weixuan Ning, Astrid A. Andersson, Ryan Ho Leung Tsang, Eric Ka Yip Liu, Billy C.H. Hau, Juha Merilä

**Affiliations:** a Conservation Forensics Laboratory, Area of Ecology and Biodiversity, School of Biological Sciences, The University of Hong Kong, Hong Kong Special Administrative Region; b Ecology, Evolution, and Organismal Biology Department, Iowa State University, Ames, IA, 50011, USA; c Agriculture, Fisheries and Conservation Department, The Government of the Hong Kong Special Administrative Region, Hong Kong Special Administrative Region

**Keywords:** Agarwood, Illegal harvesting, Sanger sequencing, Whole genome resequencing, Wildlife crime

## Abstract

Incense trees (*Aquilaria sinensis*) are illegally harvested across Asia due to the high market value of the agarwood they produce. This problem is particularly prevalent in Hong Kong, where government agencies are actively focused on reducing illegal harvesting by finding new means of deterrence and actively prosecuting criminal suspects. We conducted the first case study on incense trees in Hong Kong, where plant materials from criminal suspects' clothing were matched to an illegally harvested source tree using Sanger and whole genome resequencing approaches. We identified several plant particles collected from clothing of suspects to be from incense trees and traced them back to a specific illegally harvested tree. This evidence led to the prosecution of nine suspects who were sentenced to 30–50 months in jail under the Organized and Serious Crimes Ordinance (OSCO) in Hong Kong. This was one of the first times OSCO was used in illegal wildlife trade prosecution in Hong Kong, leading to 25% longer sentences. The result demonstrates that even small, aged and poorly preserved wood particulates can yield enough genomic DNA for whole genome analyses to be used as forensic evidence to support the prosecution of criminal suspects.

## Highlights

• Illegal harvesting of incense trees occurred in Hong Kong (HK).
• Whole genome sequence data were successfully obtained from small wood chips.
• DNA from wood chips on suspects' clothing matched DNA from illegally harvested trees.
• Suspects were successfully prosecuted with the aid of DNA evidence.
• One of the first time the Organized and Serious Crimes Ordinance is applied to a wildlife crime in HK.

## 1 Introduction

The illegal wildlife trade of plants and animals is a major global problem estimated to generate up to 23 billion US dollars annually, making it one of the most lucrative illicit businesses [1,2]. The identification and tracing of illegally sourced botanicals or their derivatives in particular, can be challenging, often requiring the application of advanced forensic techniques. While forensic techniques such as visual identification and chemical analyses have been developed to identify illegally harvested botanicals [3], species identification is difficult because of limited reference resources. Moreover, botanicals are often processed into different items, unrecognisable from the original plant [4]. Recent developments in genetics have driven the use of molecular forensic techniques in investigations of illegally traded botanicals. For instance, microsatellite markers have been used to identify illegally cut trees in Poland [5] and the geographic origin of wood samples from two highly traded timber species (i.e. *Erythrophleum* sp. in Africa [6], *Aquilaria malaccensis* in Malaysia [7]). Notably, the DNA evidence provided in Nowakowska et al. [5] led to the successful prosecution of illegal harvesters. However, till now, only a few studies have applied whole genome resequencing-based methods in wildlife forensics (e.g. *Panthera tigris* [8], *Manis* sp. [9]).

These DNA approaches have a growing relevance to sought-after botanicals like *Aquilaria sinensis* in South China and Southeast Asia, where illegal trade is devastating wild populations. *A. sinensis*, are part of a genus of trees commonly referred to as incense trees [[10], [11], [12]]. This specific species is native to South China and parts of tropical Southeast Asia [10,11]. It is best known as a primary source of agarwood — a dark, fragrant, resinous wood formed in response to fungal infection or wounding of the tree [13]. Agarwood has been highly prized for centuries in traditional Chinese medicine, religious rituals and the perfume industry, holding significant cultural, medicinal and commercial value [14,15]. Despite its economic and cultural importance, *A. sinensis* is facing mounting conservation challenges in the wild due to illegal and destructive harvesting practices [[15], [16], [17]]. As such, it is currently listed as “Vulnerable” on the IUCN Red List due to significant declines in wild populations [18,19]. *A. sinensis* is protected under CITES Appendix II (CITES; cites.org/eng/app/appendices.php), as well as by a host of local laws in specific jurisdictions [19]. In Hong Kong, illegal incense tree harvesting has been recorded every year since at least 2005, with a total of 847 reported cases between 2005 and 2023 [16,19]. The majority of illegal harvesting was conducted by poachers entering Hong Kong with visitor permits or via illicit channels [16,19]. Such criminal activity is driven by the widespread depletion of wild incense trees in Chinese Mainland and the relatively more abundant occurrence in Hong Kong [16,19]. To deter illegal harvesting in Hong Kong, the Agriculture, Fisheries and Conservation Department (AFCD) of the Hong Kong Special Administrative Region Government has implemented metallic tree guards, infrared camera traps and regular patrols to monitor incense trees in Hong Kong [19]. While these measures have reduced the number of illegal harvesting cases, illegal harvesting across Hong Kong continues [12,16,19].

Here, we describe a case study of illegal harvesting of incense trees by an organised crime syndicate, as well as the results of DNA-based forensics that helped to prosecute the criminal suspects. Using a combination of Sanger and whole genome resequencing (WGR) strategies applied to wood particulates collected from suspects’ clothing, we were able to match the DNA sequences from the latter to those of an illegally harvested tree individual, providing evidence for the successful prosecution of the criminals. We hope that the transparent, replicable genomic approach presented here will serve as a model to guide future investigations into illegal harvesting of botanicals and improve the conservation prospects of rare and threatened wildlife.

## 2 Materials and methods

### 2.1 Case of illegal incense tree harvesting in Hong Kong and evidence collection

**Fig. 1:**
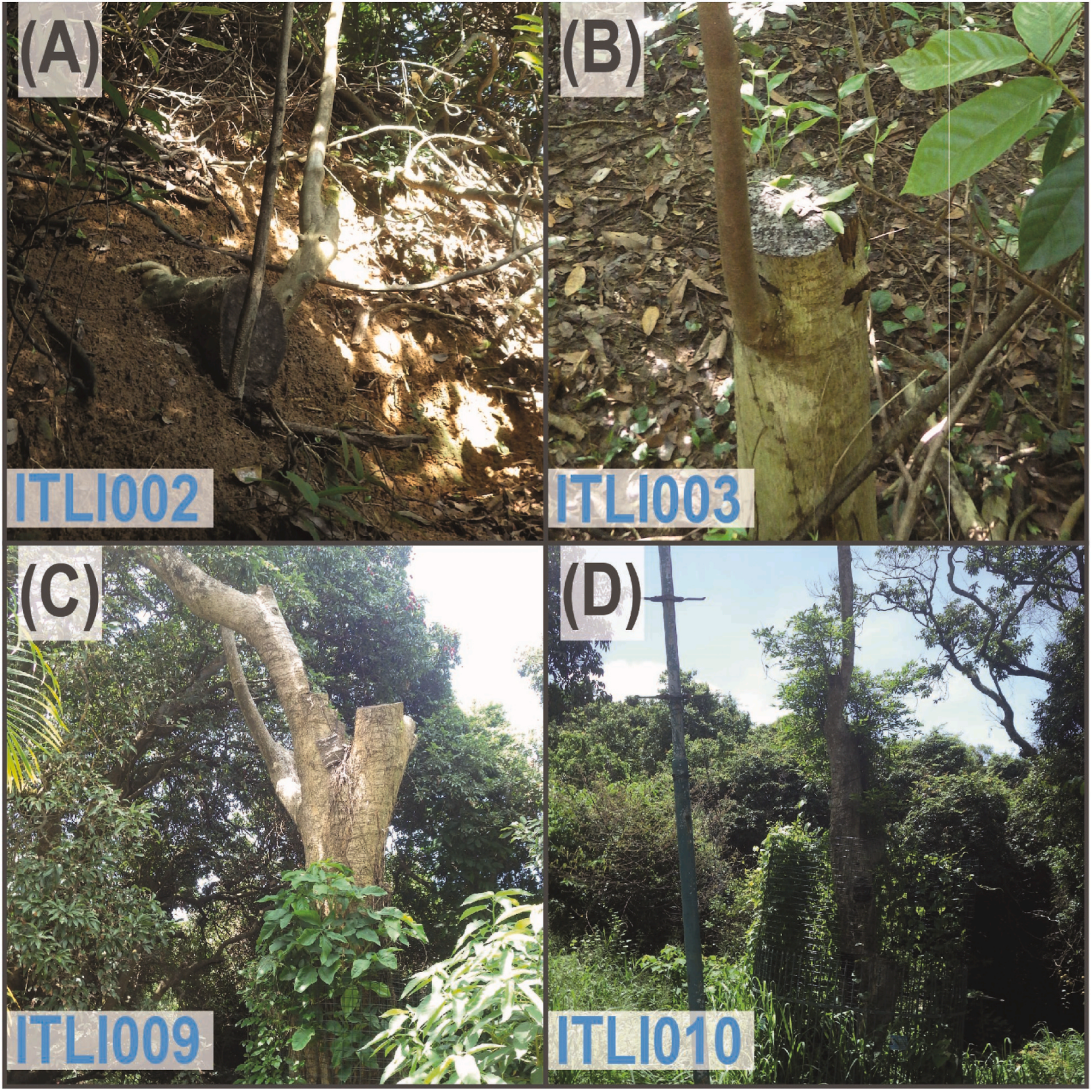
Four of the seven illegally harvested incense trees under investigation in this case with ID: (A) ITLI002, (B) ITLI003, (C) ITLI009, (D) ITLI010.

In February and March of 2023, seven *A. sinensis* trees were illegally harvested on Lamma Island in Hong Kong (Fig. 1). Thirteen individuals suspected of being part of a syndicate of incense tree harvesters were arrested. While these suspects were not arrested on the site of the illegal harvesting, the descriptions of some were reported by local eyewitnesses or caught in possession of tree-cutting tools, leading to their arrests. In addition to tree-cutting tools, personal items found on the suspects, including dirtied backpacks and clothing (i.e. shirts, gloves, shoes and pants), were confiscated and sealed in individual evidence bags by police officers. The clothing and backpacks were visually examined by three of the authors and police officials on June 5^th^, 2023, to identify and collect plant materials from them. Plant particulates dislodged from the confiscated items during handling and accumulated at the bottom of the evidence bags were also collected. The plant particulates identified included dry leaves, fragments of woody tissue (i.e. tiny chips and twigs), bark and flowers (Fig. S1). Each piece of plant material removed was photographed by both a police photographer and a member of the scientific team and sealed in sterile Eppendorf tubes. Sterile procedures were followed to avoid the possibility of cross-contamination, which included changing gloves, sterilising any equipment used and all surfaces before a new evidence bag was opened. After all samples were collected, they were stored in a secure −20°C freezer that had never contained any plant materials.

To investigate whether the suspects had indeed been involved with cutting down any of the seven Lamma Island incense trees, we collected fresh leaves and, in one case, a piece of wood from the recently felled/injured trees (i.e. those harvested between February and March 2023). We treated these samples as our potential sources, placing them individually in sealed plastic bags before transferring them to a clean laboratory at The University of Hong Kong for DNA extraction. This sampling was conducted on June 21^st^, 2023.

### 2.2 DNA extraction of plant tissue

Each plant tissue was manually ground to a fine powder by immersing samples and particulates in liquid nitrogen using a set of autoclaved pestle and mortar. DNA was extracted from each sample using the DNeasy® Plant Mini Kit (QIAGEN) following the manufacturer's recommendations. To avoid cross-contamination, separate time periods, two different laboratories and two sets of extraction kits were used to extract DNA from samples from Lamma Island (i.e. potential source trees) and evidence particulates from the confiscated personal items of suspects. In particular, DNA extractions of the particulates were performed in a clean laboratory where no *A. sinensis* extractions had ever taken place, and no other plant material had previously been present. Furthermore, we extracted and processed DNA from these particulates prior to collecting samples from the potential source trees. This was done to eliminate the risk of contamination from the fresh materials, which have a higher DNA yield than the evidence particulates. Additionally, two negative controls were included in each extraction batch to detect if any cross-contamination occurred during the extraction process. DNA products were quantified through spectrophotometry on a NanoDrop One (ThermoFisher) and Qubit 2 fluorescence (by the sequencing companies) to determine the quantity and quality of DNA extracts before sequencing.

### 2.3 Species identification of plant material through Sanger sequencing

As a provisional step to validate if any extracted materials could be matched to the *Aquilaria* genus, two commonly used plant-specific genomic regions were amplified through polymerase chain reactions (PCR) and Sanger sequenced [20]. These regions were the nuclear Internal Transcribed Spacer Region (ITS, 451 bp) and the plastid maturase *K* gene (*matK*, 682 bp). The ITS and *matK* regions were selected due to their suggested ability to identify *A. sinensis* from other *Aquilaria* species [20,21] and their wide use in botanical phylogenetics [21]. These regions were amplified following the primers and cyclic conditions as presented in Kang et al. [20]. Primer sets were synthesised by the Beijing Genomics Institute (BGI; Hong Kong) and the PCR amplifications were conducted in-house on a Bio-Rad T100 Thermal Cycler. The primer sequences and their references are provided in Table S1. The PCR amplification reactions consisted of 35 μl of total volume per reaction, including 10.5 μl of GoTaq, 7 μl of 5 nm concentration forward and 7 μl of 5 nm concentration reverse primer, 7 μl of purified water and 3.5 μl of eluted DNA extract. All PCR amplifications were done with a negative control (i.e. a reaction where all reagents were added, but lacking DNA, which was replaced with purified water) to ensure no contamination had occurred. The cycle sequencing reaction was as follows: 94°C for 3 min, 38 cycles of 94°C for 30 s, 53°C for 45 s, 72°C for 1 min, followed by a 72°C step for 10 min and a final step maintained at 4°C. The PCR results were visualised on 2% agarose gels (Fig. S2). If targeted gene amplification was observed, the PCR products were then sent for bi-directional Sanger sequencing at BGI Hong Kong (Table S2). The sequences were visualised using the free chromatogram visualisation software Ugene [22]. Herein, sequence ends were trimmed and ambiguous DNA bases were resolved by comparing and assembling the forward and reverse sequences.

To identify taxa from DNA Sanger sequences of the amplified PCR products, we performed sequence similarity searches against global databases. Specifically, we compared our sequenced results against those on GenBank (National Centre for Biotechnology Information; NCBI), which stores 1.6 billion sequences from 450,000 species, using the nucleotide BLAST algorithm. To provide further authenticity of both the sequences from evidence particulates and potential source trees blasted to *Aquilaria*, a maximum likelihood phylogeny for each amplified region was generated using MEGA6 [23] with 1000 bootstrap replicates. The phylogenies included *A. sinensis* reference sequences for ITS (from Hong Kong) and *matK* (from Chinese Mainland) in addition to those from three other *Aquilaria* species: *A*. *hirta*, *A*. *malaccensis* and *A*. *yunnanensis* (Table S3).

Lastly, Sanger sequences of particulates and samples are available on GitHub (github.com/uvafung; Table S2). Only particulates identified as *Aquilaria* (specifically *A. sinensis* from the phylogenies) among those taken from evidence, along with subsequent potential source material, were sent for next-generation sequencing.

### 2.4 Next-generation sequencing

DNA extracted from evidence-based plant particulates and fresh samples of the source trees were sent to the HKU Centre for PanorOmic Sciences (CPOS; Table S4) for whole genome resequencing. Polymerase chain reaction-based Illumina libraries were applied to DNA extracts from particulates (due to lower concentrations and possible degradation) and PCR-free Illumina libraries to the fresh samples. All evidence particulates and samples were then sequenced on the Illumina NovaSeq 6000 (CPOS) to a depth of 10× coverage.

### 2.5 Raw read data processing

Raw reads from both particulates taken from evidence items and samples from collected potential source trees were processed with the same bioinformatics pipeline. Adapters were trimmed using AdapterRemoval v2.3.1 [24]. Each specimen was mapped to the *A. sinensis* genome generated from a Hong Kong tree (GCA_011066525.1 [25]) using bwa-mem v0.7.15-r1140 [26]. The subsequent bam files were marked for duplicates using PicardTools v2.18 (broadinstitute.github.io/picard), then sorted and indexed using samtools [27]. All processing scripts are available on GitHub (github.com/uvafung). In addition, 33 previously published whole genome resequencing reads [28] representing four regions in Hong Kong were downloaded from the SRA database and were processed in the same manner following the same bioinformatics pipeline to provide broad geographic context to the study.

### 2.6 Single Nucleotide Polymorphism (SNP) calling

Given the uneven depth coverage of our data, we used Analysis of Next Generation Sequencing Data v0.940 (ANGSD [29]) to call Single Nucleotide Polymorphism (SNP) variants. ANGSD v0.940 estimates the genotype likelihoods for each individual at each genomic position. The genotype likelihood represents the probability of observing a specific genotype given the sequencing data and the underlying genetic model, taking into account the sequencing error rates, mapping quality and other relevant factors. Therefore, these genotype likelihoods provide a continuous measure of uncertainty, allowing for more nuanced and probabilistic genotype calls. After initial quality filtering, the final SNP set was further filtered in PLINK v1.90 [30] for minor allele frequency (0.05), Hardy-Weinberg Equilibrium (HWE; 1 × 10^−5^) and linkage disequilibrium and combination of samples, with the final SNP sets of 619,975 and 467,423 SNPs (available on GitHub; github.com/uvafung).

### 2.7 Genomic analyses

A Principal Component Analysis (PCA) was conducted in PLINK v2.0 [30] to identify (1) broad genetic relationships between all sampled individuals and (2) only samples collected from Lamma Island. The results were plotted in R [31] with individuals coloured by location. To identify potentially matching individuals, we calculated basic statistics among individuals (--relatedness and --kinship flags) in PLINK v1.9 [30] to provide estimates of identity by descent (IBD; z0, z1, z2 and PI_hat) and among sampling locations, as well as to determine if any specimens examined were relatives (such as parent-offspring and full-siblings). To validate our methodological approach to individual assignments using IBD, known full-sibling samples were first excluded from the bootstrap validation to avoid artificially inflating similarity and potentially confounding the match assignment. Each police item was then compared individually against the remaining tree samples. To account for sampling variance and assess the robustness of the match, pairwise IBD comparisons were repeated 100 times for each police item (i.e. bootstrap replicates), with each replicate drawing a random subset of 10,000 SNPs from the full dataset.

## 3 Results

### 3.1 Sanger sequencing identifications of evidence particulates

The Sanger sequencing of ITS and *matK* indicated that five evidence particulates from personal items (IT018/019, IT023, IT036, IT037 and IT038) could be matched to *A. sinensis*. Comparisons of these sequences to those in GenBank confirmed that these evidence particulates and those of samples taken from potential source trees had the highest blasting affinity to *A. sinensis* (Table S2). We also identified that some of the evidence particulates, not blasting to *A. sinensis*, matched other species known to be prevalent on Lamma Island (e.g. IT030 matching *Dalbergia benthamii* and IT047 matching *Psychotria asiatica*; Table S2). In addition to these results, the gene phylogenies also showed a close relationship of these particulates and sample sequences to *A. sinensis* (Fig. S3). For both the ITS and *matK* loci, all evidence and source trees identified as *A. sinensis* yielded identical Sanger sequences with no sequence variation across the amplified regions, and each sequence matched *A. sinensis* reference sequences in GenBank with 100% identity (Fig. S3).

### 3.2 Whole genome identifications of evidence particulates

**Fig. 2:**
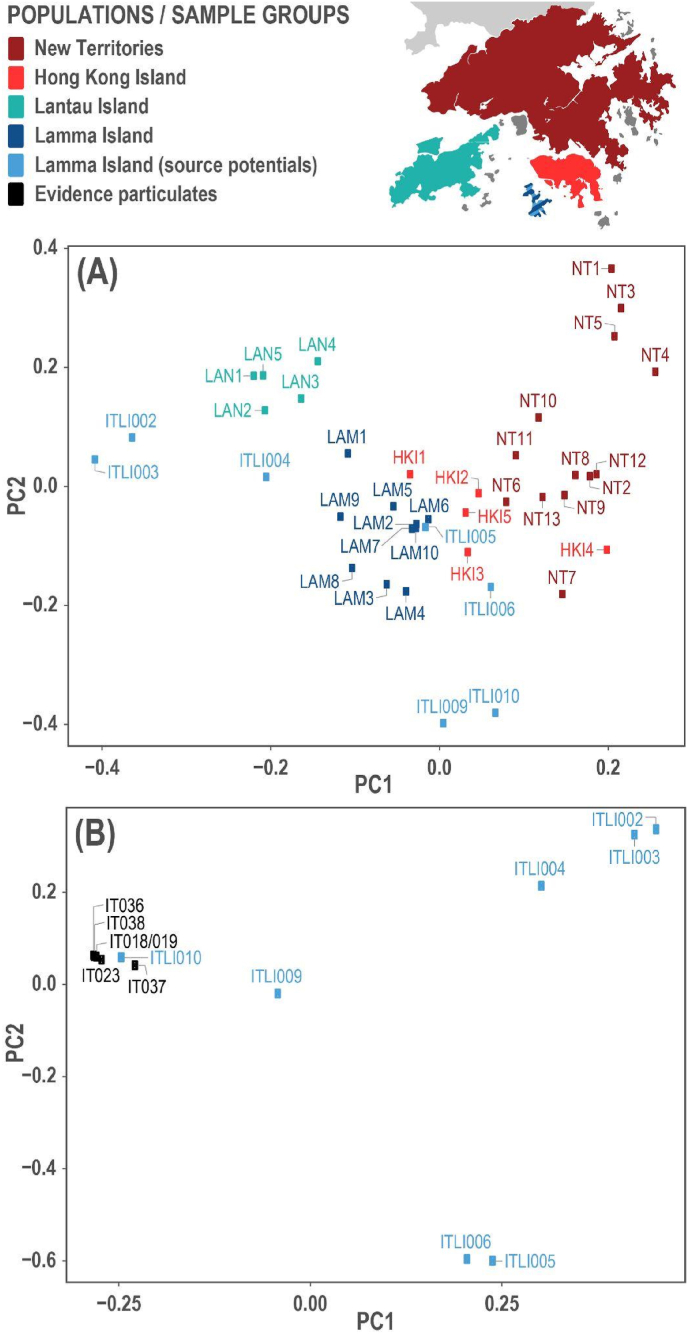
(A) A principal component analysis (PCA) of anonymised regional samples shows that the illegally harvested trees (i.e. potential sources; ITLI002, ITLI003, ITLI009, ITLI010) cluster with other trees on Lantau and Lamma Island (light blue vs dark blue and turquoise markers). (B) A PCA of samples from Lamma Island, showing the plant particulates taken from the personal items of evidence (black markers) and the potential source trees (light blue markers).

**Fig. 3:**
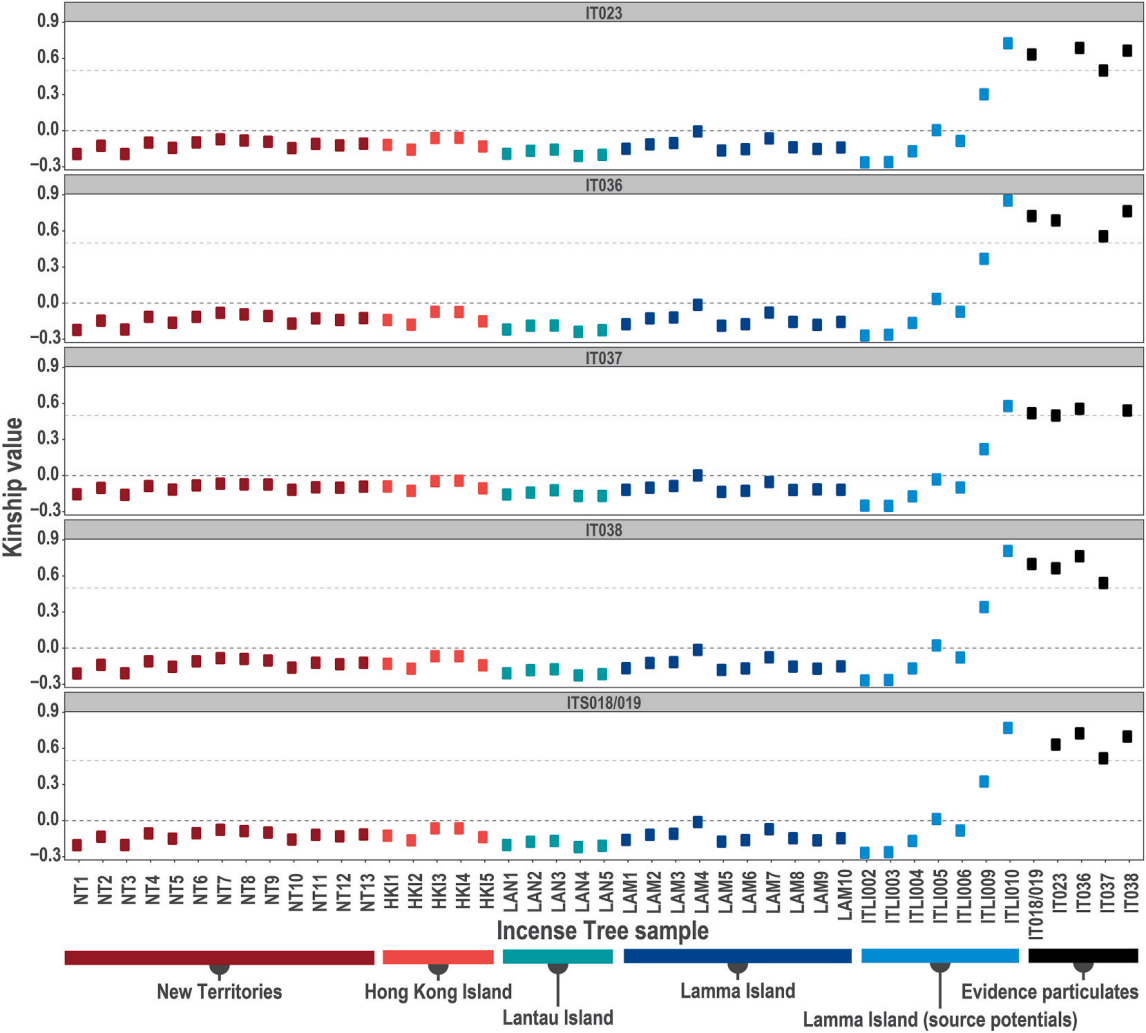
Kinship analyses comparing whole genome resequencing (WGR) profiles of evidence particulates taken from the suspects' personal items (IT023, IT036, IT037, IT038, IT018/019; black markers) to those of potential source trees (light blue markers) and other trees from Hong Kong colour-coded based on geographic region (also see key in Fig. 2). Note that tree ITLI009, growing in close proximity to the illegally harvested tree (ITLI010), was a close kin (a full-sibling) of ITLI010.

The WGR data showed that potential source trees from Lamma Island group closely with existing genomic data we had available for incense trees from Lamma Island as compared to other locations across Hong Kong in our broad PCA (Fig. 2A). Moreover, the sequences from evidence particulates obtained from the personal items of suspects, positively identified as *A. sinensis* (through Sanger sequencing), in turn grouped closely with the potential source trees from Lamma Island (Fig. 2B). This PCA grouping, shows that the genetic makeup of the *A. sinensis* evidence particulates is well within the scope of those trees originating from Lamma Island. More importantly, all particulates positively identified as *A. sinensis* were shown to derive from the same specific tree – potential source tree ITLI010 (Lamma Island) – based on the PCA and kinship analyses (Fig. 3; Table 1).

**Table 1:** Summary of Kinship coefficients for the best match, the single identified sibling match and the highest next match to potential source tree and reference trees sampled across wide geographic area in Hong Kong.

Particulates	Coverage (×)	Top match	Kinship coefficient	Sibling coefficient	Highest non-match coefficient
IT036	6.90	ITLI010	0.850	0.367	0.034
IT038	6.09	ITLI010	0.807	0.341	0.020
IT018/019	4.91	ITLI010	0.769	0.324	0.012
IT023	4.15	ITLI010	0.724	0.300	0.002
IT037	2.67	ITLI010	0.577	0.214	0.001

**Fig. 4:**
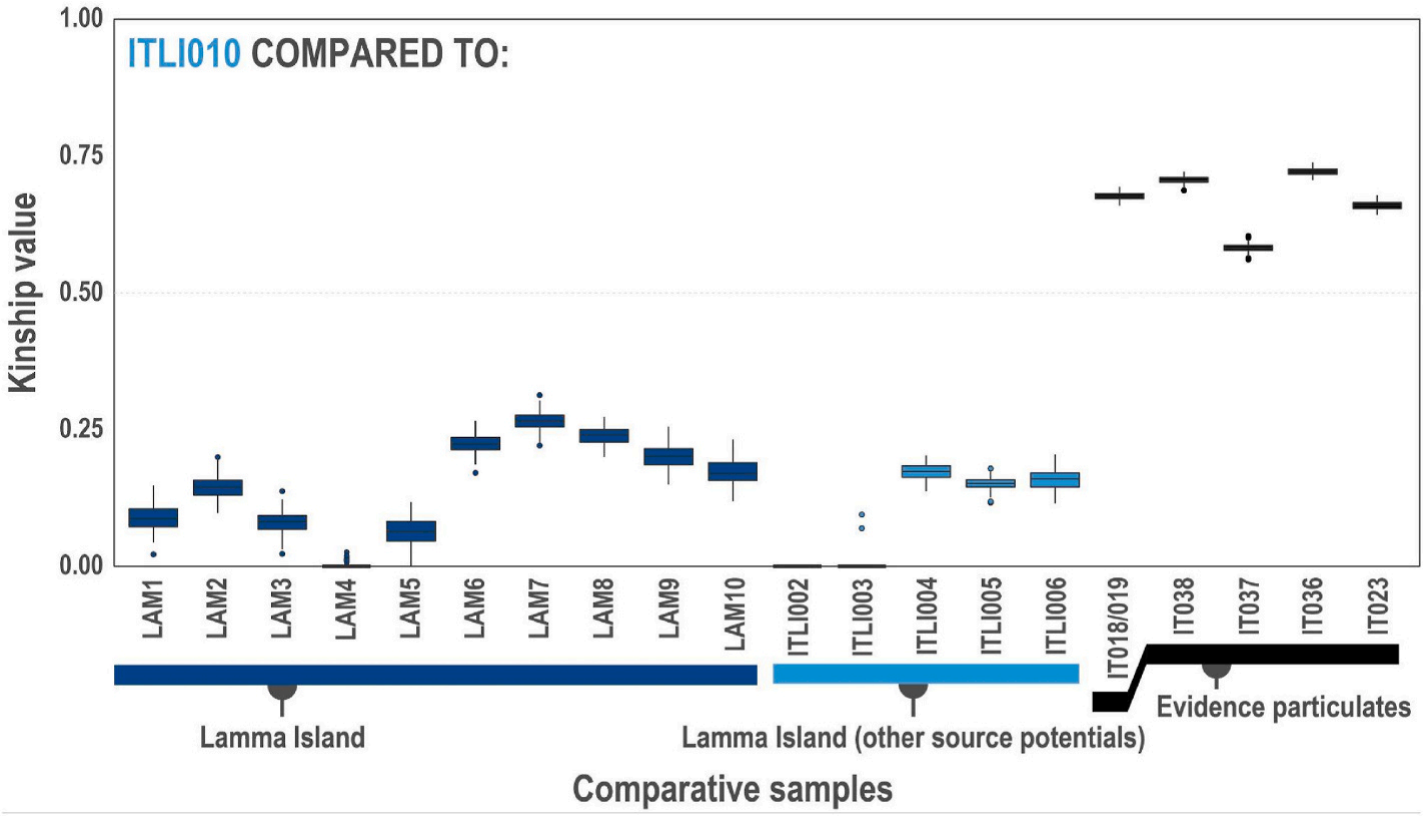
Results from 100 bootstrap replicates comparing kinship values of source tree ITLI010 to *A. sinensis* evidence particulates taken from the suspects' personal items (i.e. IT023, IT036, IT037, IT038, IT018/019; black box and whisker plots) and non-matching trees from Lamma Island (blue box and whisker plots). Kinship values are plotted to show the variation in relatedness among replicates. Tree ITLI009, a full-sibling to the illegally harvested tree (ITLI010), is omitted from this analysis.

The DNA from ITL010 matched the DNA extracted from the evidence particulates sampled from the suspects’ clothing, in each instance, as the clear singular matches, with kinship values ranging from the poorest quality sample (IT037) kinship estimate as 0.577 to the best quality sample (IT036) with a kinship value 0.850 (Fig. 3). These matches achieved in our primary analysis were supported in >99% of the bootstrap replicates, with ITLI010 being the highest and only corresponding to a full match with the *A. sinensis* evidence particulates (Fig. 4). The next closest genomic match of these evidence particulates, was that of potential source tree ITLI009 (full-sibling relationships, ranging from 0.219 for the poorest quality sample (IT037 to 0.367 for the best quality sample IT036), which grew in close proximity to ITL010 and was found to be a full-sibling of the latter (0.344; Fig. 3).

## 4 Discussion

The successful matching of the DNA extracted from evidence particulates collected from confiscated personal items to the DNA of one of the potential source trees (ITLI010) provided strong evidence leading to successful prosecution. Due to the organised nature of the crime, prevalence of the offence and the impact on local cultural and social relevance of the community, the Organized and Serious Crimes Ordinance (OSCO; Cap. 455 of the Hong Kong Legislation) was adopted in the sentencing of all nine convicts. This was one of the first times the OSCO was adopted in the sentencing of illegal wildlife crime in Hong Kong, resulting in a 25% sentence enhancement. As a result, six convicts received a 30-month jail sentence, two received a 35-month sentence and one received a 50-month sentence. Here, it is also worth noting that this case became linked to a cross-border police operation where 11 additional suspects associated with this case were successfully arrested in Guangdong for their involvement in the illegal harvesting of incense trees [32].

The workflow of our case study began with Sanger sequencing, which proved to be a fast and cost-effective method to identify the tree species of wood particulates, accomplished in the course of five days. As it turned out, not all of the wood particulates originated from incense trees, but also from other species such as *Dalbergia benthamii* and *Psychotria asiatica*. This step helped save time and resources by narrowing down the whole genome resequencing effort to only samples verified to be from incense trees.

Obtaining higher molecular weight DNA for WGR from small and poorly preserved evidence items is challenging [33,34]. In our case, the evidence items had been stored in sealed plastic bags at room temperature for ∼2 months before DNA extractions were performed. Given Hong Kong's subtropical climate with high temperature and humidity, DNA degrades rapidly and reduces the quality of DNA that can be extracted. Yet, as results show, we successfully extracted sufficient DNA to perform WGR for most samples. Hence, even small fragments of old and poorly stored material can be used to obtain enough DNA to accurately link evidence to sources.

To minimise the risk of contamination, we first processed DNA extractions from the evidence materials with lower expected DNA yield, then conducted extractions from the potential source trees in a separate laboratory. Hence, the extractions and additional processing were separated both in time and space, using non-overlapping reagents and instruments in spaces where no incense tree material had previously been handled. These measures turned out to be sufficient to avoid contamination, as all negative controls remained negative. Future forensic work would ideally be conducted in a dedicated forensics laboratory in Hong Kong or specially curated clean rooms developed for low-yield samples.

This is one of the first criminal cases in the world that uses WGR technology to provide DNA evidence in a court of law for the prosecution of criminals. Whole genome resequencing has been widely applied in various scientific fields such as population genetics, virology and public health, but its application in forensic science remains in its early stages. In the United States, whole genome sequencing evidence will be accepted for the first time in a homicide case, set to stand trial in 2026 [35]. In our case, WGR has successfully identified the best-matching tree with a high bootstrap support value, showing that our results are robust and reliable. While in an ideal population with Hardy-Weinberg equilibrium, the kinship value of two matching samples should be 1.0, in forensic cases the reality is often lower due to the need to extract DNA from poorly preserved evidence. As in this case, older, poorly stored particulates are subject to high degradation. This lower DNA quality causes the accumulation of more mismatches [12]. A higher sequencing depth could increase the accuracy of the WGR calls, in turn generating kinship values that are closer to the expected value in theory and providing stronger evidence in court, but may be challenging where the quantity of DNA extracts is limited.

While the use of WGR data provided us with a sufficient amount of data for matching different samples, we note that obtaining, handling and processing this type of data is quite time-consuming. Therefore, for future forensic work, the development of a SNP panel tailored for individual matching of sourced and evidence material would provide a faster and less cumbersome path to obtain meaningful results. Such panels have now been developed *in silico* for other species [36,37], bypassing the expensive development of traditional SNP-panels such as the Illumina Golden Gate assay (https://www.illumina.com/documents/seminars/presentations/2009_08_downing_jason.pdf, [38]) or the MassARRAY iPLEX platform [39].

As illegal harvesting of incense trees continues in Hong Kong and elsewhere [12,19], the experience gained from this case study should pave the road for similar studies should illegal harvesting incidents with similar evidence take place in future. The approach used here is also applicable to other incense tree species for which sufficient reference material is available, such as *A. malaccensis* syn *A. agallocha* and *A. yunnanensis,* which are also subject to illegal harvesting [12]. We hope that by being transparent about our methodology and approach, this case study can inspire and benefit law enforcement and the conservation of the biodiversity of tropical Southeast Asia.

## Declaration of generative AI and AI-assisted technologies in the manuscript preparation process

No generative AI and AI-assisted technologies were used in the manuscript preparation process.

## Funding sources

This work was supported by a Hong Kong Police Force contract and ACFD tender (AFCD/SQ/137/22) to the team, grants from the Research Grants Council of the Hong Kong Special Administrative Region (Project reference numbers: Seed Grant for Collaborative Research 2207101593 and Area of Excellence Grant AoE/M-702/26-R), as well as The University of Hong Kong Faculty of Science RAE Enhancement Fund 2024/2025 grant to B.C.H.H. Additionally, U.Y.Y.F. was supported by the Hong Kong PhD Fellowship and the HKU Presidential Scholarship.

## CRediT authorship contribution statement

**Kerry Reid:** Conceptualization, Formal analysis, Investigation, Methodology, Writing – original draft, Writing – review & editing. **Uva Y.Y. Fung:** Formal analysis, Visualization, Writing – original draft, Writing – review & editing. **Arthur F. Sands:** Investigation, Visualization, Writing – original draft, Writing – review & editing. **Weixuan Ning:** Investigation, Writing – review & editing. **Astrid A. Andersson:** Writing – review & editing. **Ryan Ho Leung Tsang:** Investigation, Writing – review & editing. **Eric Ka Yip Liu:** Investigation, Writing – review & editing. **Billy C.H. Hau:** Supervision. **Juha Merilä:** Conceptualization, Supervision, Writing – original draft, Writing – review & editing.

## Declaration of competing interest

The authors declare that they have no known competing financial interests or personal relationships that could have appeared to influence the work reported in this paper.

## References

[1] Nellemann C., Henriksen R., Kreilhuber A., Stewart D., Kotsovou M., Raxter P., Mrema E., Barrat S. (2016). https://wedocs.unep.org/handle/20.500.11822/7662.

[2] Lautensach and Lautensach (2020). Human Security in World Affairs: Problems and Opportunities.

[3] Dormontt E.E., Boner M., Braun B., Breulmann G., Degen B., Espinoza E., Gardner S., Guillery P., Hermanson J.C., Koch G., Lee S.L., Kanashiro M., Rimbawanto A., Thomas D., Wiedenhoeft A.C., Yin Y., Zahnen J., Lowe A.J. (2015). Forensic timber identification: it's time to integrate disciplines to combat illegal logging. Biol. Conserv..

[4] Manzanilla V., Teixidor-Toneu I., Martin G.J., Hollingsworth P.M., de Boer H.J., Kool A. (2022). Using target capture to address conservation challenges: population-level tracking of a globally-traded herbal medicine. Mol. Ecol. Resour..

[5] Nowakowska J., Oszako T., Tereba A., Konecka A. (2015).

[6] Vlam M., Arjen de Groot G., Boom A., Copini P., Laros I., Veldhuijzen K., Zakamdi D., Zuidema P.A. (2018). Developing forensic tools for an African timber: regional origin is revealed by genetic characteristics, but not by isotopic signature. Biol. Conserv..

[7] Lee S.L., Zakaria N.F., Tnah L.H., Ng C.H., Ng K.K.S., Lee C.T., Lau K.H., Chua L.S.L. (2022). DNA databases of a CITES listed species *Aquilaria malaccensis* (Thymelaeaceae) as the tracking tools for forensic identification and chain of custody certification. Forensic Sci. Int..

[8] Khan A., Krishna S.M., Ramakrishnan U., Das R. (2022). Recapitulating whole genome based population genetic structure for Indian wild tigers through an ancestry informative marker panel. Heredity.

[9] Gu T.-T., Wu H., Yang F., Gaubert P., Heighton S.P., Fu Y., Liu K., Luo S.-J., Zhang H.-R., Hu J.-Y., Yu L. (2023). Genomic analysis reveals a cryptic pangolin species. Proc. Natl. Acad. Sci. U. S. A..

[10] Ali N., Jin C.B., Jamil M., Preedy V.R. (2016). Essential Oils in Food Preservation, Flavor and Safety.

[11] Du T.-Y., Dao C.-J., Mapook A., Stephenson S.L., Elgorban A.M., Al-Rejaie S., Suwannarach N., Karunarathna S.C., Tibpromma S. (2022). Diversity and biosynthetic activities of agarwood associated fungi. Diversity.

[12] Fung UYY, Sands AF, Andersson AA, Reid K, Ning W, Tam ST, Tsang RHL, Liu EKY, Wang R, Yin J-T, Liu C-Y, Hau BCH, Merilä J**.** Genetics and protection of *Aquilaria* species (Thymelaeaceae) vulnerable to illegal harvesting. Review.

[13] Naziz P.S., Das R., Sen S. (2019). The scent of stress: evidence from the unique fragrance of agarwood. Front. Plant Sci..

[14] Al-Woozain A.M. (2016). https://interactive.aljazeera.com/aje/2016/oud-agarwood-scent-from-heaven/index.html.

[15] Thompson I.D., Lim T., Turjaman M. (2022).

[16] Jim C.Y. (2015). Cross-border itinerant illegal harvesting of agarwood in Hong Kong's peri-urban forests. Urban For. Urban Green..

[17] Chen Y., Liu H., Heinen J. (2019). Challenges in the conservation of an over-harvested plant species with high socioeconomic values. Sustainability.

[18] Wang Z.F., Cao H.L., Cai C.X., Wang Z.M. (2020). Using genetic markers to identify the origin of illegally traded agarwood-producing *Aquilaria sinensis* trees. Glob. Ecol. Conserv..

[19] AFCD (2023).

[20] Kang Y., Liu P., Lv F., Zhang Y., Yang Y., Wei J. (2022). Genetic relationship and source species identification of 58 Qi-Nan germplasms of *Aquilaria* species in China that easily form agarwood. PLoS One.

[21] Xie Z., Fan S., Xu J., Xiao H., Yang J., Guo M., Cheng C. (2025). Identification and classification of *Aquilaria* (Thymelaeaceae): inferences from a phylogenetic study based on *matK* sequences. PeerJ.

[22] Okonechnikov K., Golosova O., Fursov M., UGENE team (2012). Unipro UGENE: a unified bioinformatics toolkit. Bioinformatics.

[23] Tamura K., Stecher G., Peterson D., Filipski A., Kumar S. (2013). MEGA6: molecular Evolutionary Genetics Analysis version 6.0. Mol. Biol. Evol..

[24] Schubert M., Lindgreen S., Orlando L. (2016). AdapterRemoval v2: rapid adapter trimming, identification, and read merging. BMC Res. Notes.

[25] Nong W., Law S.T., Wong A.Y., Baril T., Swale T., Chu L.M., Hayward A., Lau D.T., Hui J.H. (2020). Chromosomal‐level reference genome of the incense tree *Aquilaria sinensis*. Mol. Ecol. Resour..

[26] Li H. (2013). Aligning sequence reads, clone sequences and assembly contigs with BWA-MEM. arXiv:1303.3997v1 [q-bio.GN].

[27] Li H., Handsaker B., Wysoker A., Fennell T., Ruan J., Homer N., Marth G., Abecasis G., Durbin R., 1000 Genome Project Data Processing Subgroup (2009). The Sequence Alignment/Map format and SAMtools. Bioinformatics.

[28] Law S.T.S., Nong W., Yip H.Y., Liu E.K.Y., Ng T.P.T., Tsang R.H.L., Xia N., Shaw P.-C., Lam H.M., Lau D.T.W., Hui J. (2023). Population genomic analyses of protected incense trees *Aquilaria sinensis* reveal the existence of genetically distinct subpopulations. Front. Ecol. Evol..

[29] Korneliussen T.S., Albrechtsen A., Nielsen R. (2014). ANGSD: analysis of next generation sequencing data. BMC Bioinf..

[30] Chang C.C., Chow C.C., Tellier L.C.A.M., Vattikuti S., Purcell S.M., Lee J.J. (2015). Second-generation PLINK: rising to the challenge of larger and richer datasets. GigaScience.

[31] R Core Team (2025). https://www.R-project.org/.

[32] HK01 (2024). ‘13內地斬樹黨砍伐南丫島土沉香被捕 10人罪成加刑囚30至50個月’ [13 Mainland Chinese individuals arrested for illegally harvesting incense trees on Lamma Island, 10 were prosecuted and sentenced to jail for 30–50 months.]. 8 November 2024. https://www.hk01.com/%E7%AA%81%E7%99%BC/1074221/13%E5%85%A7%E5%9C%B0%E6%96%AC%E6%A8%B9%E9%BB%A8%E7%A0%8D%E4%BC%90%E5%8D%97%E4%B8%AB%E5%B3%B6%E5%9C%9F%E6%B2%89%E9%A6%99%E8%A2%AB%E6%8D%95-10%E4%BA%BA%E7%BD%AA%E6%88%90%E5%8A%A0%E5%88%91%E5%9B%9A30%E8%87%B350%E5%80%8B%E6%9C%88.

[33] Staats M., Erkens R.H.J., van de Vossenberg B., Wieringa J.J., Kraaijeveld K., Stielow B., Geml J., Richardson J.E., Bakker F.T. (2013). Genomic treasure troves: complete genome sequencing of herbarium and insect museum specimens. PLoS One.

[34] Wagner S., Lagane F., Seguin-Orlando A., Schubert M., Leroy T., Guichoux E., Chancerel E., Bech-Hebelstrup I., Bernard V., Billard C., Billaud Y., Bolliger M., Croutsch C., Čufar K., Eynaud F., Heussner U.K., Köninger J., Langenegger F., Leroy F., Lima C., Martinelli N., Momber G., André Billamboz A., Nelle O., Palomo A., Piqué R., Ramstein M., Schweichel R., Stäuble H., Tegel W., Terradas X., Verdin F., Plomion C., Kremer A., Orlando L. (2018). High-throughput DNA sequencing of ancient wood. Mol. Ecol..

[35] Watson M., Morales M. (2025). https://edition.cnn.com/2025/09/02/us/rex-heuermann-murder-trial-new-dna-testing.

[36] Mamugy F., Bertola L.D., Mertens De Vry A., Dussex N., Shiffthaler B., Paijmans J.L.A., Hofreiter M., Forbes R., Kerley G.I.H., Everatt K., Becker M., Creel S., Bourgeois S., Chifunte C., Drouilly M., Spong G. (2024). SNP panel for non-invasive genotyping of leopard (*Panthera pardus*). bioRxiv 2024.11.01.621452.

[37] Zenato Lazzari G., Figueiró H.V., Sartor C.C., Donadio E., Di Martino S., Draheim H.M., Eizirik E. (2025). Development of a SNP panel for geographic assignment and population monitoring of jaguars (*Panthera onca*). Ecol. Evol..

[38] Pavy N., Gagnon F., Deschênes A., Boyle B., Beaulieu J., Bousquet J. (2016). Development of highly reliable in silico SNP resource and genotyping assay from exome capture and sequencing: an example from black spruce (*Picea mariana*). Mol. Ecol. Resour..

[39] Dormontt E.E., Jardine D.I., van Dijk K.-J., Dunker B.F., Dixon R.R.M., Hipkins V.D., Tobe S., Linacre A., Lowe A.J. (2020). Forensic validation of a SNP and INDEL panel for individualisation of timber from bigleaf maple (*Acer macrophyllum* Pursch). Forensic Sci. Int. Genet..

