# Supplementary material for: Linking suspects to an illegally felled incense tree (Aquilaria sinensis) using DNA forensics
Source: Forensic Sci Int Synerg. 2026 Aug 11;13:100721. doi: 10.1016/j.fsisyn.2026.100721 (PMC13487622; doi:10.1016/j.fsisyn.2026.100721)
Supplement: Supplementary file 1 [file mmc1.docx]

**Supplementary Materials**

**Figure S1:** Photos of evidence particulates from personal items attempted for extraction. Photos were taken by the Hong Kong Police (Marine Region), *Marine Regional Headquarters* in Sai Wan Ho, Hong Kong. As sampled particulates were being cataloged and photographed, they were given unique laboratory identification numbers and placed in sterile collection tubes.

**Table S1.** Primer sequences used for Internal Transcribed Spacer Region (ITS, 451 bp) and the plastid maturase K gene (*matK*, 682 bp) PCR amplification.

| **Region** | **Primer** | **Primer sequences (5’– 3’)** | **References** |
| --- | --- | --- | --- |
| **ITS2** | ITS-S2F | ATGCGATACTTGGTGTGAAT | Cheng et al. 2016 |
| **ITS2** | ITS-S3R | GACGCTTCTCCAGACTACAAT | Cheng et al. 2016 |
| ***matK*** | 3F_KIM | CGTACAGTACTTTTGTGTTTACGAG | Li et al. 2018 |
| ***matK*** | 1R_KIM | ACCCAGTCCATCTGGAAATCTTGGTTC | Li et al. 2018 |

**Figure S2.** PCR products from plant material collected from (A) particles on personal items (see 5.1) and (B) from fresh tissue and wood pieces (see 5.2) for the Internal Transcribed Spacer Region (ITS, 451 bp) and the plastid maturase K gene (*matK*, 682 bp), respectively. When PCR amplification is successful, a dark band can be seen on the gel in the size range of the expected fragment size given the ladder that is run. In these gels, the dark bands at 450 bp for the ITS region and above 650 bp for the *matK* gene can be observed. Whereas for samples with extremely weak bands or negative controls lacking bands entirely, amplification was not successful.

**Table S2.** A summary of collected evidence particulates with the sample code, type of biological material, polymerase chain reaction success for each targeted gene region, closest GenBank match (by genus and species), sequences are available in FASTA format at (link excluded for anonymity). Not every sample with PCR amplification could be successfully sequenced.

| **Sample/Particulate code** | **Biological material** | **ITS** | ***matK*** | **GenBank ID**  **(Genus)** | **GenBank ID**  **(Species)** |
| --- | --- | --- | --- | --- | --- |
| **IT030** | leaf | yes | yes | *Dalbergia* | *benthamii* |
| **IT032** | stem | no | no | *No amplification* | *No amplification* |
| **IT037** | wood chips | yes | yes | *Aquilaria* | *sinensis* |
| **IT043** | bark | no | no | *No amplification* | *No amplification* |
| **IT018/019** | wood chips | yes | yes* | *Aquilaria* | *sinensis* |
| **IT023** | wood chips | yes | yes* | *Aquilaria* | *sinensis* |
| **IT036** | wood chips | yes | yes | *Aquilaria* | *sinensis* |
| **IT038** | wood chips | yes | yes | *Aquilaria* | *sinensis* |
| **IT041** | leaf | yes | no | *Psychotria* | *asiatica* |
| **IT046** | stick | no | no | *No amplification* | *No amplification* |
| **IT047** | stick | yes | no | *Psychotria* | *asiatica* |

*Although amplification was positive in gel electrophoresis, Sanger sequencing was unsuccessful.

**Table S3.** GenBank sequence accession numbers and information of *Aquilaria hirta*, *Aquilaria malaccensis* and *Aquilaria yunnanensis* used in the construction of ITS and *matK* phylogenies.

| **GenBank Accession** | **Species** | **Genomic region** | **Geographic Region** | **Hyperlink** |
| --- | --- | --- | --- | --- |
| OM475716.1 | *Aquilaria sinensis* | ITS | Hong Kong | <https://www.ncbi.nlm.nih.gov/nuccore/OM475716.1> |
| OM909007.1 | *Aquilaria hirta* | ITS | Brunei | <https://www.ncbi.nlm.nih.gov/nuccore/OM909007.1> |
| MH134142.1 | *Aquilaria malaccensis* | ITS | Malaysia | <https://www.ncbi.nlm.nih.gov/nuccore/MH134142.1> |
| MH134148.1 | *Aquilaria yunnanensis* | ITS | Yunnan | <https://www.ncbi.nlm.nih.gov/nuccore/MH134148.1> |
| KT779120.1 | *Gyrinops ledermannii* | ITS | Indonesia | <https://www.ncbi.nlm.nih.gov/nuccore/KT779120.1> |
| OM939118.1 | *Aquilaria sinensis* | *matK* | China | <https://www.ncbi.nlm.nih.gov/nuccore/OM939118.1> |
| KY927321.1 | *Aquilaria hirta* | *matK* | Malaysia | <https://www.ncbi.nlm.nih.gov/nuccore/KY927321.1> |
| MG910414.1 | *Aquilaria malaccensis* | *matK* | Indonesia | <https://www.ncbi.nlm.nih.gov/nuccore/MG910414.1> |
| MW940123.1 | *Aquilaria yunnanensis* | *matK* | China | <https://www.ncbi.nlm.nih.gov/nuccore/MW940123.1> |
| NC_065044.1 | *Gyrinops ledermannii* | *matK* | Indonesia | <https://www.ncbi.nlm.nih.gov/nuccore/NC_065044.1> |

**Table S4.** Whole genome sequencing mapping results. The percent mapping is the percentage of sequenced reads that map to the *A. sinensis* reference genome. Coverage refers to the average number of times (×) the genome was sequenced.

| **Sample/Particulate code** | **Type** | **% mapping** | **Coverage (×)** |
| --- | --- | --- | --- |
| **IT037** | Evidence particulates | 19.8 | 2.67 |
| **IT018/019** | Evidence particulates | 45 | 4.91 |
| **IT023** | Evidence particulates | 32.4 | 4.15 |
| **IT036** | Evidence particulates | 94.8 | 6.9 |
| **IT038** | Evidence particulates | 87.7 | 6.09 |
| **ITLI002** | Potential source tree | 96.5 | 10.94 |
| **ITLI003** | Potential source tree | 99.25 | 18.08 |
| **ITLI004** | Potential source tree | 99.07 | 18.19 |
| **ITLI005** | Potential source tree | 99.24 | 14.69 |
| **ITLI006** | Potential source tree | 99.23 | 13.59 |
| **ITLI009** | Potential source tree | 95.24 | 11.32 |
| **ITLI010** | Potential source tree | 99.28 | 16.75 |

**Figure S3.** Maximum Likelihood phylogeny of (A) Internal Transcribed Spacer Region (ITS) and (B) plastid maturase K gene (*matK*), including three additional species of *Aquilaria* (*A. hirta*, *A. malaccensis* and *A. yunnanensis*) as well as a closely related outgroup *Gyrinops ledermannii*. Both phylogenies show that DNA from plant particles on personal items (labelled starting with IT and indicated in green with brackets) group with known *A. sinensis* from the potential source trees from Lamma Island (indicated in blue) and GenBank *A. sinensis* samples with high (87 – 99%) probability. Sample A1 is a wood piece taken as evidence, as found next to two poached trees on Lamma, and its PCR product was also Sanger sequenced for the *matK* region. A genomic library for this sample could not be prepared and its subsequent inclusion in downstream analyses was abandoned.

**Supplementary Materials References**

**Cheng T, Xu C, Lei L, Li C, Zhang Y, Zhou S. 2016.** Barcoding the kingdom Plantae: new PCR primers for *ITS* regions of plants with improved universality and specificity. *Molecular Ecology Resources* 16: 138–149.

**Li Q, Yan H, Lin D, Wang Y, He M, Zhang W, Gao X, Zhu S. 2018.** Molecular Identification of Three *Aquilaria* (Thymelaeaceae) Species through DNA Barcoding. *Biological and Pharmaceutical Bulletin* 41(6): 967–971.
