# Supplementary material for: Linking suspects to an illegally felled incense tree (Aquilaria sinensis) using DNA forensics
Source: Forensic Sci Int Synerg. 2026 Aug 11;13:100721. doi: 10.1016/j.fsisyn.2026.100721 (PMC13487622; doi:10.1016/j.fsisyn.2026.100721)
Supplement: Supplementary file 2 [file mmc2.docx]

Processing scripts, Sanger sequenced data in fasta format and genome-wide SNP datasets used in this study are available on the GitHub repository: (link excluded for anonymity)
